# Cryoanalgesia in Lung Transplantation – A Systematic Review and Meta-analysis

**DOI:** 10.1016/j.jhlto.2025.100263

**Published:** 2025-04-08

**Authors:** Felipe S. Passos, Pedro B. Bregion, Rachid E. Oliveira, Thierry Siemeni, Ricardo E. Treml, Bernardo M. Pessoa, Hristo Kirov, Torsten Doenst, Shaf Keshavjee, Tulio Caldonazo

**Affiliations:** a Department of Thoracic Surgery, INCAR Hospital, Santo Antônio de Jesus, Brazil; b State University of Campinas, São Paulo, Brazil; c Department of Thoracic Surgery, Barretos Cancer Hospital, Barretos, Brazil; d Department of Cardiothoracic Surgery, Jena University Hospital, Jena, Germany; e Department of Anesthesiology, Perioperative and Pain Medicine, Stanford School of Medicine, California; f Department of Thoracic Surgery, University of Alberta, Edmonton, Canada; g Division of Thoracic Surgery, Toronto Lung Transplant Program, Toronto General Hospital, University Health Network, University of Toronto, Toronto, Canada; h Department of Cardiothoracic Surgery, Weil Cornell Medicine, New York

**Keywords:** Cryoanalgesia, Epidural analgesia, Standard of care analgesia, Lung transplantation

## Abstract

**Background:**

Lung transplantation is a crucial treatment for end-stage lung diseases. However, postoperative pain management remains a significant challenge. Therefore, this study aims to examine the implications of adoption cryoanalgesia on lung transplantation pain control protocol.

**Methods:**

Three databases were searched for studies comparing cryoanalgesia versus standard of care analgesia in patients after lung transplantation. The primary outcome was opioid consumption throughout the entire hospitalization, at postoperative day (POD) 7 and at POD 14 addressed with Morphine Milligram Equivalents (MME). The secondary outcomes were maximum reported pain score at POD 7, hospital length of stay (LOS) and time until extubation. Mean differences (MDs) with 95% confidence intervals (CIs) were calculated for continuous outcomes.

**Results:**

A total of 5 studies encompassing 485 patients undergoing lung transplantation were included, of whom 228 underwent cryoanalgesia. Compared to standard of care, cryoanalgesia demonstrated significant reduction in opioid consumption at POD 7 (MD: −96.79 MME, 95% CI −183.40 to −10.18, p=0.03), at POD 14 (MD −225,26 MME; 95% CI −366.58 to −83.94; p<0.01) and throughout the entire hospitalization (MD: −307.76 MME, 95% CI −461.72 to −153.79, p<0.01). In addition, there was a significant reduction in pain scores in the cryoanalgesia group (MD: −1.10 points, 95% CI −1.77 to −0.43, p<0.01). However, no significant differences were found regarding hospital LOS or time until extubation.

**Conclusions:**

This meta-analysis indicates that cryoanalgesia effectively reduces opioid requirements and pain levels in lung transplant patients.

## Graphical abstract

**Figure:**
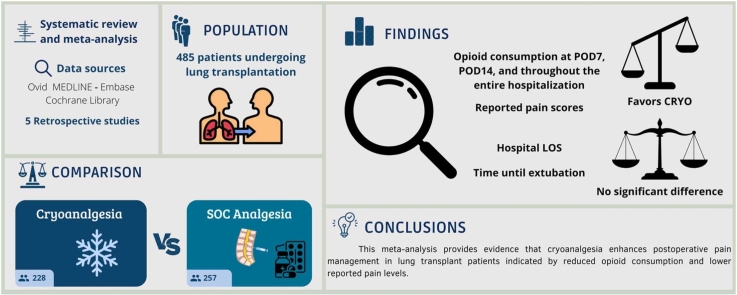

## Introduction

Lung transplantation is a critical therapeutic intervention for patients with various end-stage lung diseases, including chronic obstructive pulmonary disease (COPD), cystic fibrosis, and pulmonary fibrosis.^1^, ^2^ Over the past decades, substantial advancements in donor selection, organ preservation, perioperative management, and postoperative care have significantly improved patient outcomes.^3^, ^4^ Despite these advancements, challenges persist, particularly in managing the postoperative pain associated with the surgical procedure.^5^ This is especially relevant when using the Clamshell incision, a bilateral transverse thoracosternotomy, frequently employed in these procedures, which causes considerable discomfort due to the extensive disruption of thoracic structures.^6^ Alternatively, bilateral anterolateral sternum-sparing thoracotomy (BAT) may reduce musculoskeletal trauma, potentially improving pain control and recovery.^7^, ^8^

Effective pain management is essential, as inadequate control of postoperative pain can lead to increased morbidity and prolonged recovery times.^9^ Although opioids continue to be the cornerstone of postoperative pain management, their associated side effects, including the risks of abuse, misuse, and addiction, are well-documented.^10^, ^11^ To optimize pain relief, a multimodal analgesia approach is often implemented. While these methods have demonstrated efficacy, their impacts are limited by their relatively short duration of action.^5^ Additionally, the presence of anatomical abnormalities and concerns related to coagulopathy, infection, and hemodynamic compromise often restrict their use in patients who undergo lung transplantation.^12^, ^13^

Among the emerging pain management strategies, cryoanalgesia has garnered a promising technique. This method involves the targeted cooling of specific nerves to reversibly inhibit peripheral nerve function, providing pain relief that can last from weeks to months.^9^ Cryoanalgesia has a long history of use in alleviating chronic nerve pain and providing analgesia for surgically exposed nerves. However, data regarding its application in thoracic surgery remain limited, with most studies focusing on posterolateral thoracotomy and minimally invasive repair of pectus excavatum.^14^, ^15^ The precise effects and implications of cryoanalgesia in the context of lung transplantation have yet to be thoroughly investigated and understood.

Therefore, this systematic review and meta-analysis aims to evaluate the clinical outcomes associated with cryoanalgesia in lung transplant patients, with the goal of possibly enhancing pain management strategies and improving overall patient care.

## Methods

The study selection followed the Preferred Reporting Items for Systematic Reviews and Meta-Analyses (PRISMA) guidelines.^16^ The review was registered in the International Prospective Register of Systematic Reviews (PROSPERO, CRD42024582139).

### Search strategy

A comprehensive literature search was performed on Ovid MEDLINE, EMBASE and the Cochrane Library to identify contemporary studies comparing outcomes between cryoanalgesia and standard care analgesia in patients following lung transplantation, published up to November 2024. Additionally, we searched for additional studies using the references of previously included studies. The complete search strategy is available in Supplemental Table 1.

### Study selection

Two independent reviewers (FP and PB) screened the records after deduplication. Discrepancies between reviewers were resolved through discussion and consensus, with final decisions made by a third author (TC). Titles and abstracts were reviewed against pre-defined inclusion and exclusion criteria.

### Eligibility criteria

Inclusion criteria for studies involved in this analysis were as follows: (I) randomized controlled trials (RCTs) or observational studies; (II) comparing cryoanalgesia versus the institution’s standard of care in lung transplantation; (III) enrolling adult patients undergoing lung transplantation; and (IV) reporting at least one outcome of interest. Exclusion criteria included studies involving animal models, conference abstracts, case reports, and non-comparative study designs.

### Quality assessment and publication bias

The quality of included studies was assessed using the Cochrane Collaboration tool for assessing the risk of bias in non-randomized studies (ROBINS-I).^17^ In this assessment, each study was categorized as critical, serious, moderate, or low risk in the seven domains: confounding, selection, classification, deviations from intended interventions, missing data, and selection of reported results. Publication bias was assessed for the primary outcome.

### Data extraction

Two reviewers (FB and PB) independently performed data extraction. Accuracy was verified by a third author (TC). The extracted variables included study characteristics (publication year, time frame, country, sample size, intervention and control groups and reported outcomes) as well as patient demographics (age, sex, lung allocation score, type of transplant, and type of incision).

### Outcomes

The primary outcomes were total opioid consumption, measured in Morphine Milligram Equivalents (MME), at postoperative days (POD) 7, POD 14, and throughout the entire hospitalization. Secondary outcomes included the maximum reported pain score at POD 7, time to extubation, and hospital length of stay (LOS).

### Statistical analysis

Mean Difference (MD) with 95% confidence intervals (CI) were calculated for continuous outcomes. Heterogeneity was assessed with Cochran Q test and I^2^ statistic; p<0.10 and I^2^>50% were considered significant for heterogeneity.^18^ A p-value <0.05 was considered for significant difference between the two groups. Restricted Maximum Likelihood (REML) random effects models were chosen to account for variability between studies and to offer a more generalized estimate of effect size. This approach was applied to all analyzed endpoints.^19^ A leave-one-out sensitivity analysis was performed for the primary endpoint to assess the impact of individual studies on the overall results, thereby ensuring the robustness of the findings. The Cochrane Handbook for Systematic Reviews of Interventions was used for data handling and conversion.^20^ All statistical analyses were performed using Software R, version 4.4.0 (R Foundation for Statistical Computing, Vienna, Austria).

## Results

### Study characteristics

**Figure 1:**
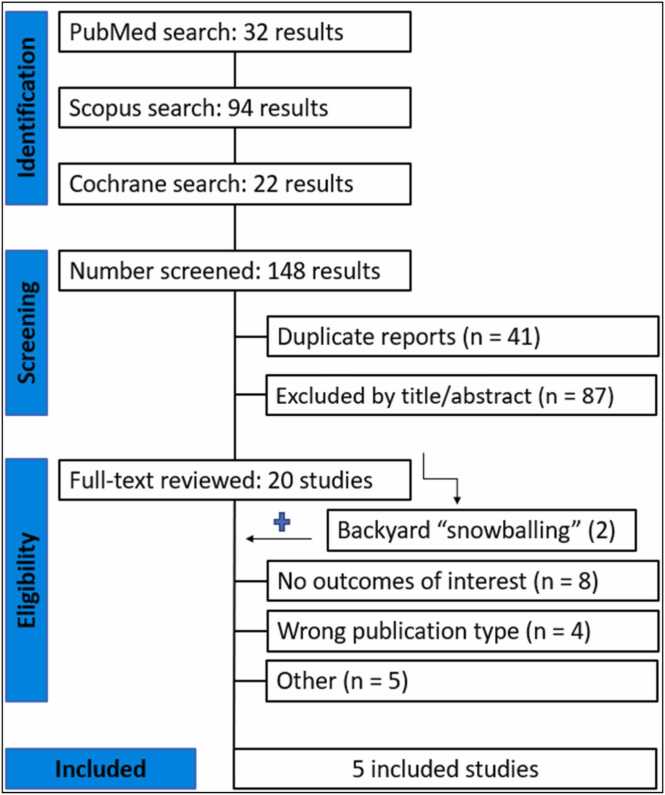
Preferred Reporting Items for Systematic Reviews and Meta-Analyses (PRISMA) flow diagram.

Figure 1 shows the PRISMA flow diagram outlining the study selection process. A total of 148 studies were retrieved from the systematic search, of which 5 met the criteria for inclusion in the final analysis.^6^, ^21^, ^22^, ^23^, ^24^ Included studies were published between 2022 and 2024. All studies used registry data originated from the United States.

### Quality assessment and publication bias

The ROBINS-I tool was utilized to assess the risk of bias, with the judgments of two independent reviewers (Supplemental Figure 1). The evaluation of bias risk across the studies included in this meta-analysis indicates a moderate level of bias, with certain domains showing lower risk.

### Patient characteristics

Table 1 shows the individual study information. Five retrospective studies were included in this meta-analysis, encompassing 485 patients. Among these, 228 patients received cryoanalgesia, while 257 underwent the institution's standard care. The number of patients in each study ranged from 52 to 170. The age ranged from 54.8 to 66 years, with the percentage of male patients varying from 53.1% to 72.5%.

**Table 1:** Study and Patient’s Baseline Characteristics of Included Studies

Study	Time frame	Country	intervention	Control	Sample size cryo/ control, n	Male cryo/ control, n (%)	Age cryo/ control, median or mean	Lung allocation score, median	Transplant type, single/ bilateral, n (%)	Type of incision, cryo/ control, n (%)^a^
Isaza 2023	2016 - 2018	USA	Cryoanalgesia	TEA	29/ 43	19 (65.5)/24 (55.8)	66/ 58*	49/ 44*	0(0)/ 72 (100)	Clamshell: 29 (100)/ 43 (100)
Kleiboeker 2024	2016 - 2021	USA	Cryoanalgesia and additional opioids or TEA as needed	Opioids and selective use of TEA	49/ 40	29 (72.5)/33 (67.3)	60/ 62*	36.6/ 37.7*	23 (25.84)/66 (74.16)	Clamshell: 38 (100)/ 28 (100)
Koons 2022	2017 - 2018	USA	Cryoanalgesia	SOC analgesia	45/ 57	25 (55.6)/35 (61.4)	65/ 61.4*	42.4/ 48.3*	23 (22.5)/79 (77.5)	NR
Pourak 2024	2020 - 2022	USA	Cryoanalgesia	TEA	20/ 32	14 (70)/17 (53.1)	55.1/ 54.8^b^	NR	0(0)/ 52 (100)	BAT: 20 (100)/ 32 (100)
Salan-Gomez 2024	2016 - 2022	USA	Cryoanalgesia	Opioids	85/ 85	53(62)/ 56 (66)	62/ 62*	40/ 39*	49 (28.82)/ 121 (71.18)	BAT: 25 (29.4)/ 24 (28.2) Clamshell: 59 (69.4)/ 59 (69.4) IBT: 1 (1.2)/ 2 (2.4)

NR: Not reported. BAT: Bilateral anterior thoracotomy; IBT: Isolated bilateral thoracotomies; SOC: Standard of care; TEA: Thoracic epidural analgesia

* Median;

a The data includes only patients who underwent bilateral lung transplantation;

b Mean.

### Primary outcome

**Figure 2:**
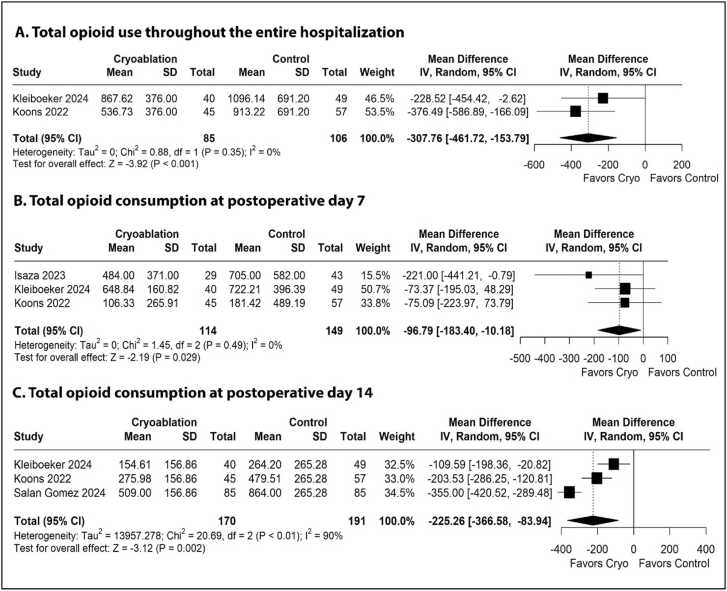
Forest plots comparing use of opioid between cryoanalgesia and standard of care analgesia for patients underwent lung transplantation. (A) Total opioid use throughout the entire hospitalization. (B) Total opioid consumption at postoperative day 7. (C) Total opioid consumption at postoperative day 14.

Table 2 summarizes the meta-analysis findings, highlighting a significant reduction in opioid consumption among patients who received cryoanalgesia. Opioid use was significantly lower throughout the entire hospitalization (MD: −307.76 MME, 95% CI −461.72 to −153.79, p<0.01, I²=0%, Figure 2A), at POD 7 (MD: −96.79 MME, 95% CI −183.40 to −10.18, p=0.03, I²=0%, Figure 2B), and at POD 14 (MD: −225.26 MME, 95% CI −366.58 to −83.94, p<0.01, I²=90%, Figure 2C). A leave-one-out sensitivity analysis for opioid use at POD 7 revealed that the overall effect size lost statistical significance when individual studies were excluded (Supplemental Figure 2). The funnel plot for this outcome did not reveal significant asymmetry (Supplemental Figure 3).

**Table 2:** Summary of Outcomes

Outcome	Number of studies	Number of patients	Effect estimate, random model (95% CI, p-value)
Total opioid consumption at POD 7	3	263	MD −96.79; 95% CI −183.40 to −10.18; p=0.03; I²=0%
Total opioid consumption at POD 14	3	361	MD −225,26; 95% CI −366.58 to −83.94; p<0.01; I²=0%
Total opioid consumption throughout the entire hospitalization	2	191	MD −307.76; 95% CI −461.72 to −153.79; p<0.01; I²=90%
Maximum pain score in a related 0−10 scale	2	174	MD −1.10; 95% CI −1.77 to −0.43; p<0.01; I²=0%
Hospital LOS	5	485	MD 2.83; 95% CI −2.10 to 7.77; p=0.26; I²=64%
Time to extubation	2	174	MD 1.75; 95% CI −1.84 to 5.33; p=0.34; I²=80%

CI: confidence interval; MD: Mean difference; POD: Post-operative day; LOS: Length of stay

### Secondary outcomes

**Figure 3:**
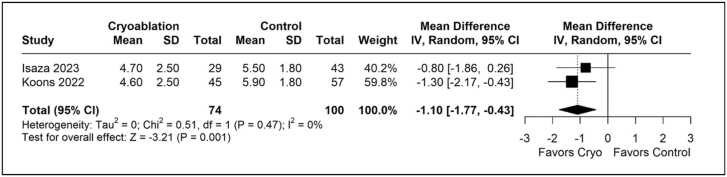
Forest plots comparing pain score reported in a 0–10 scale between cryoanalgesia and standard of care analgesia for patients underwent lung transplantation.

The cryoanalgesia group reported lower pain scores (MD: −1.10 points, 95% CI −1.77 to −0.43, p<0.01, I²=0%, Figure 3) compared to the control group.

**Figure 4:**
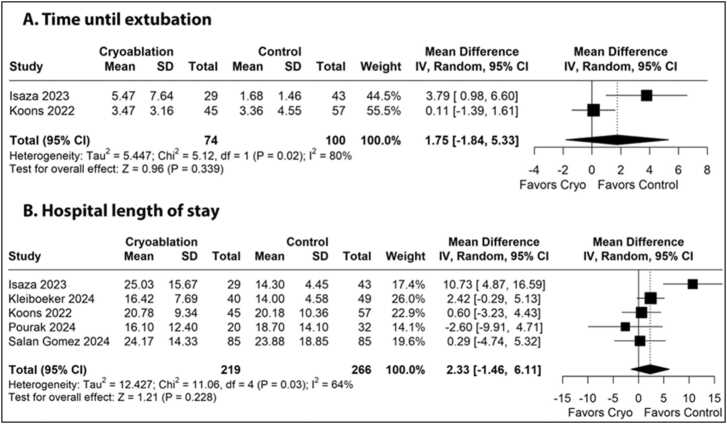
Forest plots of comparison between cryoanalgesia and standard of care analgesia for patients underwent lung transplantation. (A) Time until extubation. (B) Hospital length of stay.

Figure 4A illustrates the forest plot for time until extubation, revealing no significant difference between the cryoanalgesia and control groups (MD: 1.75 days, 95% CI −1.84 to 5.33, p=0.34, I²=80%). Figure 4B shows the forest plot for hospital LOS with no significant difference detected between the cryoanalgesia and control groups (MD: 2.33 days; 95% CI −1.46 to 6.11; p=0.23, I²=64%).

## Discussion

In this systematic review and meta-analysis of five studies including 485 patients, we comprehensively analyzed the outcomes of cryoanalgesia compared to standard analgesia techniques in patients undergoing lung transplantation. Our main findings were as follows: (I) patients receiving cryoanalgesia demonstrated a significant reduction in opioid consumption at POD 7, at POD 14, and throughout the entire hospitalization; (II) patients receiving cryoanalgesia reported lower pain scores; (III) there was no significant difference in the duration of mechanical ventilation; and (IV) there was no significant difference in hospital LOS.

Lung transplantation often represents the last opportunity for patients with end-stage lung disease to enhance their quality of life and prolong survival.^1^, ^3^, ^25^ However, postoperative pain remains a significant concern in this population, as it can interfere with the primary objectives of the transplantation procedure, leading to adverse clinical outcomes and negatively impacting overall quality of life.^5^ In this context, cryoanalgesia has emerged as a promising alternative for managing postoperative pain, particularly due to its notable duration of analgesia, which can extend from weeks to months.^9^, ^26^ However, pain management may require a tailored approach, as no single strategy is optimal for all patients. Patient-specific characteristics, such as baseline pain sensitivity, surgical complexity, response to analgesic agents, and the chosen incision type for the transplantation may influence outcomes. Additionally, variability in the number of intercostal spaces treated, cryoablation device used, and the use of adjunctive pain control techniques across centers may affect cryoanalgesia's effectiveness.

In our study, patients who underwent cryoanalgesia exhibited a marked decrease in opioid consumption at POD 7, at POD 14, and throughout the entire hospitalization. These findings align with the growing body of literature, as described by Park et al^9^ and O’Connor et al,^27^ indicating that cryoanalgesia can effectively minimize opioid requirements, addressing one of the significant challenges in postoperative care for lung transplant patients. These observations are particularly noteworthy given the increasing recognition of the complications associated with opioid use, which can hinder recovery. As highlighted by Wu et al^28^ and Benyamin et al,^11^ dependence and sedation are among the most prevalent side effects associated with opioid consumption. Therefore, minimizing opioid use aligns with current trends toward opioid-sparing strategies in postoperative care.^23^ However, it is important to acknowledge that lung transplant recipients experience pain from multiple sources, including ECMO cannulation sites, chest tube size and location, rib fractures, and type of surgical incision, which could influence the effectiveness of any analgesic strategy. Additionally, cryoanalgesia may not be universally beneficial, as factors like individual pain thresholds, pre-existing neuropathic conditions, and prior thoracic surgeries can influence its efficacy. Regarding the surgical incision for lung transplantation, the classic Clamshell incision offers superior exposure to both pleural cavities, but it is often associated with higher postoperative pain and increased risk of sternal instability and infection.^29^, ^30^ More recently, the BAT approach has garnered attention for its potential to offer adequate surgical access while potentially reducing postoperative pain and complications, primarily due to its preservation of sternal integrity.^30^ However, in our study, it was not possible to assess the specific impact of cryoanalgesia in patients undergoing Clamshell or BAT incisions due to the lack of available data in the current literature.

The lower reported pain scores in the cryoanalgesia group further support the efficacy of this technique. Reduced pain levels can enhance patient comfort and improve the efficacy of rehabilitation techniques, contributing to improved overall patient outcomes.^31^ As demonstrated by Abidi et al,^4^ although patients show significant improvements in pulmonary function following transplantation, their quality of life remains restricted by several factors, including skeletal muscle weakness. The effectiveness of exercise-based rehabilitation programs relies heavily on patient cooperation, and it is well established that adequate pain control can lead to better adherence to rehabilitation protocols and more pronounced results. Therefore, clarifying the clinical impact of cryoanalgesia beyond pain management is essential, as it may also improve functional recovery and quality of life.

The analysis of additional secondary endpoints produced no significant results in our meta-analysis. No significant differences were detected in time until extubation and hospital LOS between the cryoanalgesia and control groups. These findings suggest that while cryoanalgesia may effectively manage pain and mitigate opioid use, it does not appear to correlate directly with expedited recovery metrics. Possible reasons for these findings might include limitations in the available data, variability in patient populations, and differences in institutional protocols that influence postoperative recovery. For instance, the decision to extubate can be influenced by multiple clinical factors, such as lung function, comorbidities, and surgical complexity, which may not have been fully accounted for in this analysis.

Additionally, exploring the sources of heterogeneity within the studies is crucial. Variations in cryoanalgesia techniques, differences in patient characteristics and the absence of standardization of control groups could significantly impact outcomes and influence the overall interpretation of the data.

However, it is crucial that clinicians consider patient-specific factors when determining the optimal pain management strategy. This includes baseline pain levels, individual responses to analgesics, and potential contraindications to cryoanalgesia. While cryoanalgesia provides prolonged pain relief, concerns regarding long-term sensory changes, potential hyperalgesia, chronic allodynia and delayed recovery of nerve function must be considered.^32^, ^33^ Recommendations for implementing cryoanalgesia in clinical practice could include guidance on patient selection criteria and strategies for integrating cryoanalgesia into existing pain management protocols for lung transplant patients.

One of the most significant limitations of the current analysis is the absence of robust data on long-term outcomes associated with cryoanalgesia. Potential sequelae, such as chronic pain, neuropathic pain, and hyperalgesia, have not been evaluated in the included studies. This limitation is noteworthy given that cryoanalgesia involves ablation of multiple intercostal nerve levels, and it is known that nerve regeneration can occur with potential for the development of hyperalgesia.

Future research should focus on long-term outcomes associated with cryoanalgesia in this context, examining its effects on patient quality of life, functional recovery, and the potential impact on chronic opioid use following discharge. Additionally, further investigation is needed to assess the specific effects of cryoanalgesia in patients undergoing Clamshell or BAT incisions, as data on this aspect remain limited. By addressing these areas, we can better understand the full clinical implications of cryoanalgesia and further enhance postoperative care for lung transplant patients.

### Study strength and limitations

This is the first meta-analysis to explore the role of cryoanalgesia in lung transplantation, addressing not only opioid consumption but also secondary endpoints such as pain scores, time to extubation, and hospital LOS. Despite the encouraging outcomes, this study has some limitations. The observational nature of the included studies and their confinement to a single geographic region may limit the generalizability of the findings. Furthermore, due to inherent limitations in meta-analysis methodology, a comprehensive evaluation of potential complications related with cryoanalgesia could not be undertaken.

Additionally, the studies showed no standardization in the intervention and control groups. Variations in cryoanalgesia techniques and standard care protocols among institutions introduces a degree of variability that could affect outcomes. High heterogeneity was observed for secondary endpoints, raising questions about the consistency of the findings. Given these limitations, it is essential to interpret the results with caution. Future randomized controlled trials are essential to further validate these findings, assess its long-term impact, and clarify its role in lung transplant pain management. By standardizing interventions and employing rigorous methodologies, subsequent studies can help reduce heterogeneity and improve the reliability of evidence regarding the effectiveness of cryoanalgesia.

## Conclusion

This meta-analysis provides evidence that cryoanalgesia enhances postoperative pain management in lung transplant patients indicated by reduced opioid consumption and lower reported pain levels.

## Funding

TC was funded by the Deutsche Forschungsgemeinschaft (DFG, German Research Foundation) Clinician Scientist Program OrganAge funding number 413668513, by the Deutsche Herzstiftung (DHS, German Heart Foundation) funding number S/03/23 and by the Interdisciplinary Center of Clinical Research of the Medical Faculty Jena.

## Disclosures

None.

## Declaration of Competing Interest

The authors declare that they have no known competing financial interests or personal relationships that could have appeared to influence the work reported in this paper.

## Data Availability

The data underlying this article are available in the article and in its online supplementary material.

## Footnotes

Appendix A Supplemental data associated with this article can be found in the online version at doi:10.1016/j.jhlto.2025.100263.
