# Supplementary material for: Cryoanalgesia in Lung Transplantation – A Systematic Review and Meta-analysis
Source: JHLT Open. 2025 Apr 8;8:100263. doi: 10.1016/j.jhlto.2025.100263 (PMC12022505; doi:10.1016/j.jhlto.2025.100263)
Supplement: Supplementary file 1 — Supplemental material [file mmc1.docx]

**Supplementary Material**

**Supplementary Table 1**. Complete search strategy.

**Supplementary Figure 1.** Critical appraisal of studies according to Risk Of Bias In Non-randomized Studies of Interventions (ROBINS-I)

**Supplementary Figure 2.** Leave-one-out analysis for the primary endpoint (opioid consumption at post-operative day 7).

**Supplementary Figure 3.** Funnel plot for the primary endpoint (opioid consumption at post-operative day 7)**.**

**Supplementary Table 1.** Complete search strategy

| Search: **("cryoablation" OR "cryo analgesia" OR "cryoanalgesia" OR "intercostal ablation") AND ("epidural analgesia" OR epidural OR "standard analgesia") AND ("lung" OR "lung transplant" OR "lung transplantation" OR "thoracotomy" OR "thoracic surgery" OR "lung resection")**  ("cryoablation"[All Fields] OR "cryo analgesia"[All Fields] OR "cryoanalgesia"[All Fields] OR (("intercostal"[All Fields] OR "intercostally"[All Fields] OR "intercostals"[All Fields]) AND ("ablate"[All Fields] OR "ablated"[All Fields] OR "ablates"[All Fields] OR "ablating"[All Fields] OR "ablation"[All Fields] OR "ablational"[All Fields] OR "ablations"[All Fields]))) AND ("epidural analgesia"[All Fields] OR ("epidural"[All Fields] OR "epidurally"[All Fields] OR "epidurals"[All Fields] OR "epiduritis"[All Fields]) OR "standard analgesia"[All Fields]) AND ("lung"[All Fields] OR "lung transplant"[All Fields] OR "lung transplantation"[All Fields] OR "thoracotomy"[All Fields] OR "thoracic surgery"[All Fields] OR "lung resection"[All Fields]) |
| --- |
| **Translations** |
| **epidural:** "epidural"[All Fields] OR "epidurally"[All Fields] OR "epidurals"[All Fields] OR "epiduritis"[All Fields]  **cryoablarion:** ("cryoablation" OR "cryo analgesia" OR "cryoanalgesia" OR "intercostal ablation") AND ("epidural analgesia" OR epidural OR "standard analgesia") AND ("lung" OR "lung transplant" OR "lung transplantation" OR "thoracotomy" OR "thoracic surgery" OR "lung resection") |


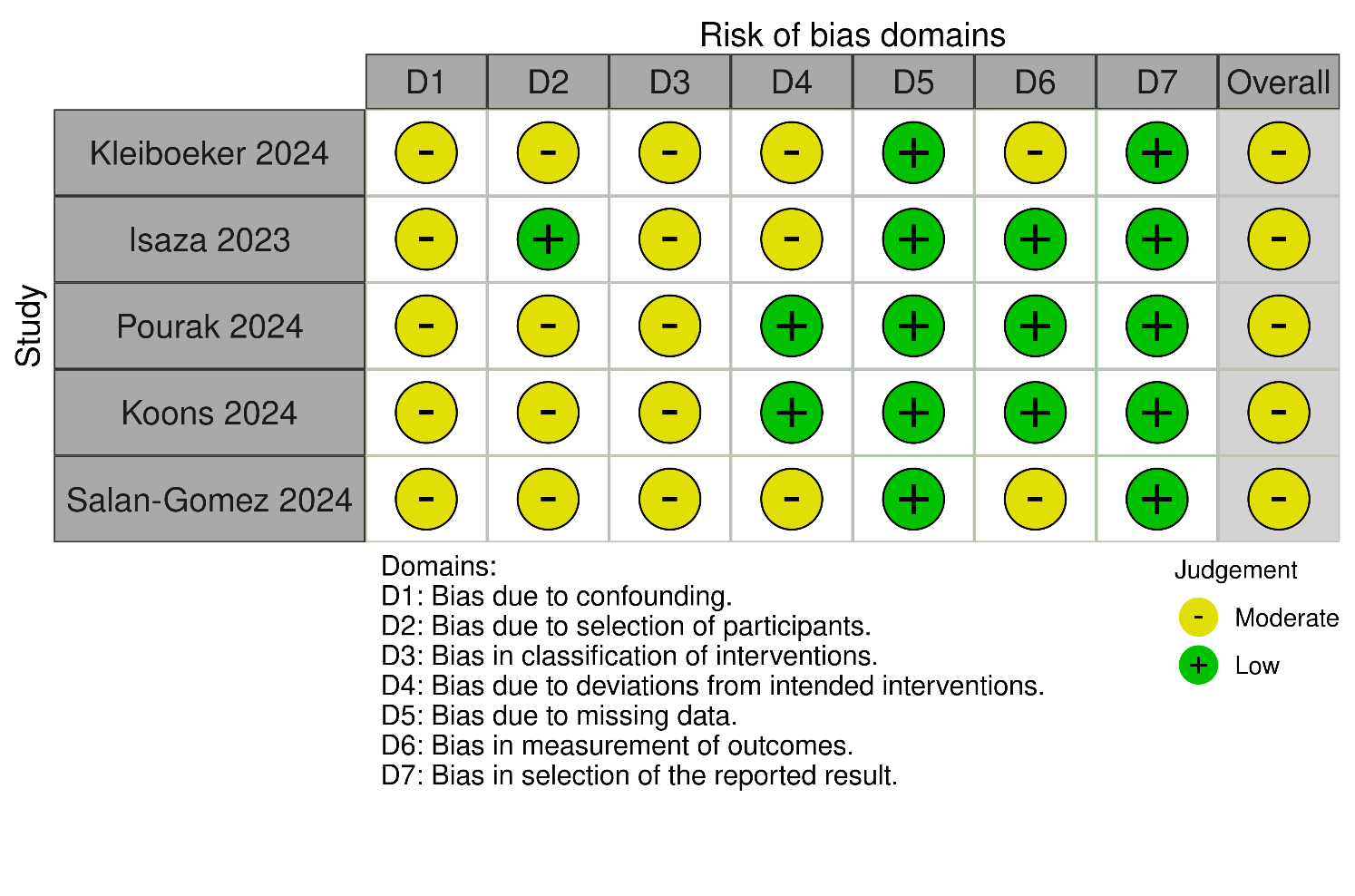
**Supplementary Figure 1.** Critical appraisal of studies according to Risk Of Bias In Non-randomized Studies of Interventions (ROBINS-I)

**
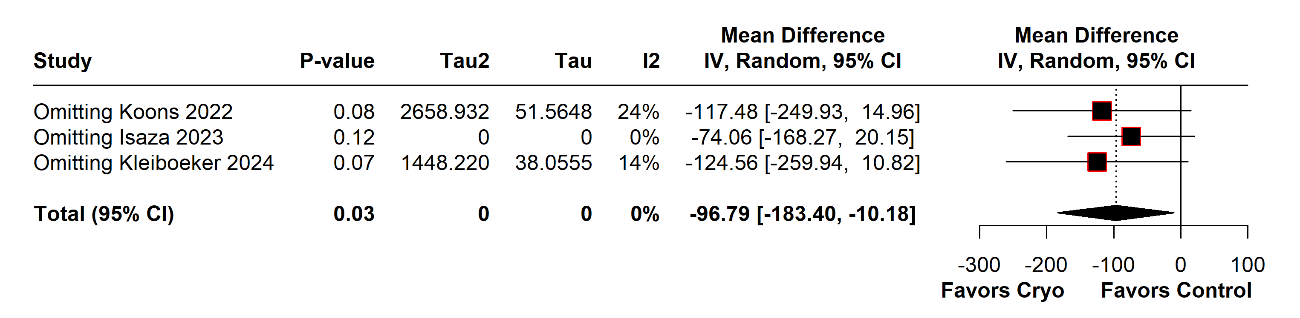
Supplementary Figure 2.** Leave-one-out analysis for the primary endpoint (opioid consumption at post-operative day 7).


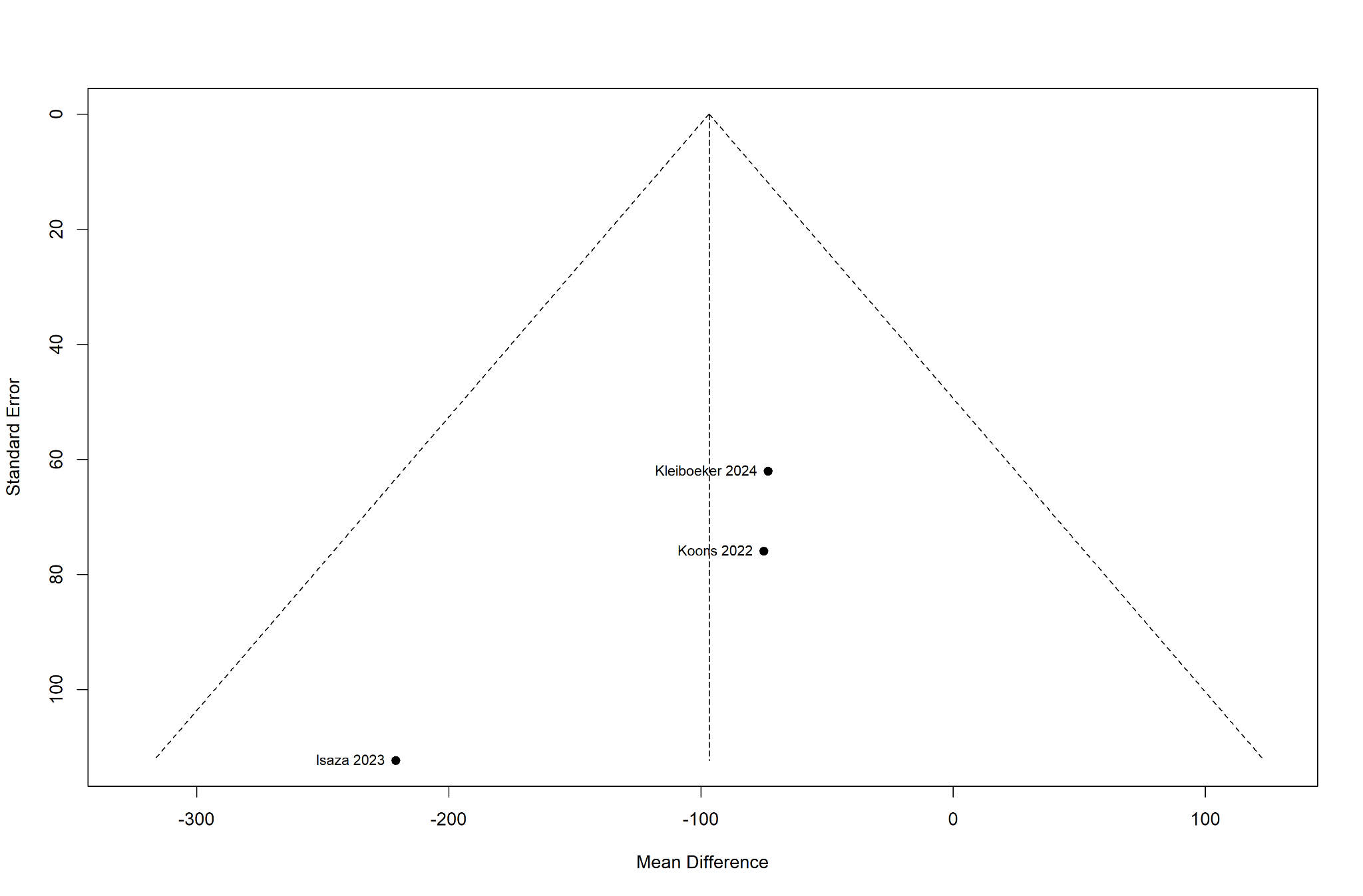
**Supplementary Figure 3 -** Funnel plot for the primary endpoint (opioid consumption at post-operative day 7).
